# Pulsed Field Ablation to Treat Atrial Fibrillation: A Review of the Literature

**DOI:** 10.3390/jcdd9040094

**Published:** 2022-03-24

**Authors:** Antonio Di Monaco, Nicola Vitulano, Federica Troisi, Federico Quadrini, Imma Romanazzi, Valeria Calvi, Massimo Grimaldi

**Affiliations:** 1 Department of Cardiology, General Regional Hospital “F. Miulli”, 70021 Bari, Italy; nicola.vitulano@gmail.com (N.V.); federica.troisi@libero.it (F.T.); federico.quadrini@tin.it (F.Q.); fiatric@hotmail.com (M.G.); 2 Department of Clinical and Experimental Medicine, University of Foggia, 71122 Foggia, Italy; 3 Department of Cardiology, Policlinico “G. Rodolico”—Azienda O.U. Policlinico “G. Rodolico”—San Marco, 95125 Catania, Italy; romanazzi.imma@gmail.com (I.R.); valcalvi@unict.it (V.C.)

**Keywords:** atrial fibrillation, catheter ablation, pulsed field ablation

## Abstract

Atrial fibrillation (AF) is the most common sustained cardiac arrhythmia and catheter ablation, which can be used in symptomatic patients refractory to antiarrhythmic therapy. Pulmonary vein isolation (PVI) remains the cornerstone of any ablation procedure. A major limitation of current catheter ablation procedures is important to recognize because even when the PVI is performed in highly experienced centers, PVI reconnection was documented in about 20% of patients. Therefore, better technology is needed to improve ablation lesions. One of the novelties in recent years is pulsed filed ablation (PFA), a non-thermal energy that uses trains of high-voltage, very-short-duration pulses to kill the cells. The mechanism of action of this energy consists of creating pores in the myocardiocyte cell membrane in a highly selective and tissue-specific way; this leads to death of the target cells reducing the risk of damage to surrounding non-cardiac tissues. In particular during the animal studies, PVI and atrial lines were performed effectively without PV stenosis. Using PFA directly on coronary arteries, there was no luminal narrowing, there has been no evidence of incidental phrenic nerve injury, and finally, PFA has been shown not to injure esophageal tissue when directly applied to the esophagus or indirectly through ablation in the left atrium. The aim of this review is to report all published animal and clinical studies regarding this new technology to treat paroxysmal and persistent AF.

## 1. Introduction

Atrial fibrillation (AF) is the most common sustained cardiac arrhythmia, occurring in 1–2% of the general population. Patients affected by this arrhythmia have an increased risk of stroke and heart failure, with a significant reduction in functional capacity and quality of life. The European guidelines recommend catheter ablation of AF in symptomatic patients refractory to drugs or with heart failure [1,2]. The pathogenesis of AF is complex and several studies reported that pulmonary vein (PV) foci play an important role in both initiation and perpetuation of this arrhythmia. Pulmonary vein isolation (PVI) remains the cornerstone of all ablation procedures irrespective of patient characteristics. Beyond PVI, various ablation strategies have been proposed into clinical practice, although scientific evidence from randomized studies was poor. These strategies include ablation of complex fractionated atrial electrograms, linear lesions, ablation of low-voltage areas, ablation of autonomic ganglia, as well as identification and ablation of rotational activities and putative AF trigger sites [3,4,5]. Although there is less evidence supporting ablation in non-paroxysmal AF, one trial has demonstrated in patients affected by persistent AF a more effective maintenance of sinus rhythm and better quality of life after ablation [6]. A recent European Survey reported that about one-third of AF ablations were performed in patients with persistent or long-standing persistent AF [7].

The conventional approach used by cardiac electrophysiologists to perform AF ablation is radiofrequency (RF) energy. There are many crucial factors to consider to determine optimal lesion size and depth after RF ablation: power, tissue impedance and temperature, ablation duration, and contact force [8,9,10]. In the clinical practice, other technologies are actually used to perform catheter ablation as cryoenergy and laser energies, and many multicentric trials have shown no differences in efficacy between all technologies [11]. However, it is important to recognize a major limitation of current catheter ablation procedures. In particular, even when the PVI is performed in highly experienced centers, PVI reconnection was documented in about 20% of patients [12]. Moreover, these technologies are associated with lesions of the organs adjacent to the heart, in particular the esophagus and phrenic nerve [1,3]. Therefore, more effective and safe technology is needed to improve ablation lesions to treat AF. One of the novelties in recent years is pulsed filed ablation (PFA), a non-thermal energy that uses trains of high-voltage, very-short-duration pulses to kill the cells [13]. The mechanism of action of this energy consists of creating pores in the myocardiocyte cell membrane in a highly selective and tissue-specific way; this leads to death of the target cells, reducing the risk of damage to surrounding non-cardiac tissues such as the esophagus, nerves, and vessels.

The aim of this review is to report all published animal and clinical studies regarding this new technology to treat paroxysmal and persistent atrial fibrillation. 

## 2. Pulsed Field Ablation: Animal Studies

Pulsed field ablation application in the clinical setting found rational basis in several laboratory studies. The safe and effective PFA delivery was tested in animal and human cell studies. Two in vitro ablation models of single-cell systems and monolayer cell systems were used to perform PFA for myocardial cell H9C2 and smooth muscle cell A7r5 [14]. Three Bama minipigs were used to verify the in vivo ablation effect of PFA. In the monolayer cell system, H9C2 was significantly sensitive to PFA compared with A7r5. Bidirectional PFA performed on Bama minipigs was able to effectively obtain PVI without stenosis; furthermore, bidirectional PFA was able to significantly ablate myocardial cells, maintain cell–cell connection, and reduce muscle contraction.

In another study [15], different types of cellular preparations were tested with different electric field strengths and pulse numbers (pheochromocytoma cells derived from Rattus norvegicus adrenal glands, a somatic cell hybrid of rat embryonic dorsal root ganglion, mouse neuroblastoma, and human neuroblastoma cells). In this study, the ablation threshold in a cell suspension model was at least 1000 V/cm with 50 pulses of 100 µs duration for both neuronal-like cells and cardiomyocytes, and slightly higher for cardiac fibroblasts. The authors suggested the importance of understanding the optimal threshold of irreversbile electroporation to achieve a safe ablation modality without affecting the nearby tissues.

One of the severe complications of PVI using thermal ablation is esophageal damages, in particular, atrio-esophageal fistula. In 84 New Zealand rabbits, nonthermal irreversible electroporation was directly applied to their esophagi. After 16 weeks from ablation, no lumen stenosis, epithelial erythema, erosion, ulcer, or fistula were reported [16]. 

A systematic review of the preclinical evidence was performed from three sources (PubMed, EMBASE, and Google Scholar), analyzing 16 animal studies assessing efficacy and safety of irreversible electroporation (five were acute suvival studies, ten were chronic survival studies, and one study involved both acute and chronic models) [17]. Irreversible electroporation was applied to the ventricular myocardium, atrial tissue/pulmonary veins, coronary arteries, esophagus, phrenic nerve, and cardiac ganglia. In total, 320 ablations were performed. Histologically, the larger and more likely transmural lesions were linked to a higher energy, a higher amplitude of pulses or longer pulses, or a greater number of pulses applied in the ventricular myocardium and atrial tissue. The ablation of ganglia was effective in 83% of animal models tested, while the vessels (coronary arteries) were generally unaffected with no signs of ulceration or other adverse reactions documented regarding the esophagi.

In a pilot study [18], it was shown that cardiac cells are significantly more susceptible to damage due to electroporation than either esophageal smooth muscle cells or neurons; moreover, the lesions caused by electroporation have a robust formation when the shocking electrode pair is in contact with cells (block threshold = 400 ± 50 V/cm), but higher field strengths are required when the electrode pair is moved away from the cells (block threshold = 690 ± 60 V/cm). 

Yavin et al. [19] reported data regarding a lattice-tip catheter (Affera Inc., Newton, MA, USA) able to erogate focal RF or PFA. The effects of PFA were analyzed in 25 swine and the authors examinated the feasibility to create an atrial line of block, and examined the block durability and phrenic nerve and esophagus damages. PFA produced acute block in 100% of the lines, and histological analysis showed 100% transmurality with a lesion width of 19.4 mm (10.9–27.4). PFA selectively affected cardiomyocytes but spared blood vessels and nervous tissue. PFA did not cause esophageal or phrenic nerve injury as opposed to RF.

Koruth at al. [20] compared the safety and feasibility of durable PVs and superior vena cava (SVC) isolation between RF ablation and PFA (PFA ablation catheter: Farapulse, Inc.; Menlo Park, CA, USA). Bipolar PFA was performed with monophasic and biphasic waveforms. All targeted veins were successfully isolated and the biphasic waveform induced significantly less skeletal muscle engagement with a higher durability of pulmonary vein isolation. Pulmonary vein narrowing and nerve damage was documented only in the RF group.The phrenic nerve was spared in all groups, but the authors found an incomplete SVC encirclement with RF. 

Yavin H et al. [21] investigated a PFA system with a circular multielectrode catheter (PFA lasso). This study included 16 swine and investigated the feasibility to create lines, the lesion durability after a 30-day survival period, and the effects of high-intensity PFA on the esophagus and phrenic nerve. Acute line of block was achieved in all swine, ablation line durability was 91.7%, PFA was transmural in 97.8% of sections, and high-intensity PFA had no effect on the esophagus and phrenic nerve. 

All the other relevant animal studies are summarized in Table 1. All the studies, despite different electrode configurations, reported data on the efficacy and safety of this new technology. 

## 3. Pulsed Field Ablation: Clinical Studies

Initial experience of PFA ablation was performed in 22 patients with symptomatic paroxysmal AF, using a monophasic waveform, either with an endocardial or epicardial ablation catheter, but no safety or efficacy follow-up data are available [36]. In particular, this study was performed at two centers and reported data regarding 15 endocardial and 7 epicardial AF ablations. PVI was obtained in 100% of patients and surgical box lesions in 86% of patients.

The IMPULSE, PEFCAT, and PEFCAT II are three trials that included 121 patients with symptomatic paroxysmal AF [37,38]. They are prospective, non-randomized, single-arm safety and feasibility trials. In these studies, a 12-F over-the-wire PFA ablation catheter (Farawave, Farapulse Inc., Menlo Park, CA, USA) was used. In the IMPULSE trials, 40 patients underwent ablation with monophasic waveforms between 900 and 1000 V under general anesthesia and paralytic agents. Biphasic waveforms between 1800 and 2000 V were used in the PEFCAT and PEFCAT II trials under conscious sedation with good tolerability. After 75–90 days from index ablation, 110 patients underwent remapping of the left atrium with a multielectrode catheter to evaluate possible reconnection of pulmonary veins. Patients were followed for 1 year by clinical visit, trans-telephonic monitor, and 24 h Holter to assess the recurrence of atrial fibrillation. The rate of primary safety events was low (2.5%) due to two cases of cardiac tamponade; there was no evidence of esophageal injury, phrenic nerve lesions, or brain lesions after ablation. All PVs were isolated by catheter, receiving a mean of 7.2 ± 2.2 ablations lesions per PV. No reconnection was observed after a 20 min waiting period or adenosine testing. After three months, remapping of the left atrium demonstrated durable PVI in 84.8% of PVs. This included 45.2% of PVs in 11 patients treated with monophasic energy, 83.6% and 96% in patients treated with biphasic energy, and with optimized biphasic protocol, respectively. After 1 year of follow up, the Kaplan–Meier estimate for freedom from AF was 81.1 ± 3.8%. In 49 patients treated with optimized biphasic protocol, the Kaplan–Meier estimate for freedom from AF was 84.6 ± 6%. 

A lattice-tip ablation catheter (Sphere-9; Affera, Inc., Watertown, MA, USA) was another catheter used in a first-in-human trial [39]. This is a 7.5F bidirectional deflectable catheter with an expandable 9 mm diameter nitinol lattice electrode, containing nine mini-electrodes (0.7 mm diameter each) on the spherical surface. The lattice tip contains a central indifferent electrode and two additional ring electrodes located just proximally on the shaft. The catheter can deliver RF and PFA, and its generator (HexaGen and HexaPulse, Affera, Inc., Newton, MA, USA) has a dual design as to permit a switch between two energies. Pulmonary vein isolation was performed with a strategy of either PFA posteriorly and RF anteriorly (RF/PF), or PFA throughout (PF/PF). Seventy-six patients with symptomatic paroxysmal or persistent AF were enrolled. The ablation energy used was RF/PF in 40 patients and PF/PF in the remaining 36 patients, with PF used on the posterior LA because of the esophagus. In persistent AF patients, additional linear ablation of the posterior mitral isthmus or the LA roof was performed per operator discretion. The primary safety end point rate was 1.3% (one patient with vascular access injury): there was no pericardial tamponade, phrenic nerve injury, brain lesions, or esophageal injury. We have not evaluated the efficacy of this catheter after 1 year of follow up. 

De Potter et al. [40] reported the first experience of PFA using a variable decapolar irrigated loop circular catheter designed to work with Carto system (Biosense Webster, Irvine, CA, USA). Thirty-five patients with symptomatic paroxysmal AF included in the INSPIRE Trial have been successfully treated. The INSPIRE trial is a prospective, non-randomized, multicentre study, planned to enrol up to 550 patients. Acute procedural success was achieved in 100% of study subjects, and initial results have demonstrated acute safety and effectiveness of the new catheter.

Other catheters are undergoing clinical evaluation for PFA. A recent study published by Verma et al. [41] reported data regarding the PULSED AF pilot trial. This first-in-human pilot phase evaluated the feasibility and efficacy of PVI using a PFA system delivering bipolar, biphasic electrical fields through a circular multielectrode array catheter (PulseSelect; Medtronic, Inc., Minneapolis, MN, USA). In this multicentric trial (six centers in Australia, Canada, the United States, and the Netherlands), the authors enrolled 38 patients affected by paroxysmal or persistent AF. The primary endpoint was the ability to achieve acute pulmonary vein isolation intraprocedurally and safely after 30 days from the procedure. The acute electrical PVI was obtained in 100% of patients and no serious adverse events occurred 30 days after procedures, including phrenic nerve injury, esophageal injury, stroke, or death.

Cochet H et al. [42] investigated the extra-atrial damages after atrial fibrillation ablation using both PFA and RF. This study reported data regarding cardiac magnetic resonance obtained pre-ablation within 3 h and 3 months after procedures in 41 patients with paroxismal AF undergoing PVI using PFA (18 patients ablated using Farapulse system) or thermal ablation (16 radiofrequency ablations and 7 cryoablations). No phrenic nerve injuries were documented. Acutely, RF and cryoablation energies caused 43% of esophageal lesions in the area of direct contact between the atrium and esophagus, while PFA did not cause esophageal lesions. Acute lesions were documented on the descending aorta in 43% of patients after thermal ablation, and in 33% after PFA. At 3 months, all lesions resolved without significant clinical complications.

Nakatani Y et al. [43] used cardiac magnetic resonance to analyze the left atrial structural and mechanical characteristics after PFA and RF ablation. This study reported data regarding cardiac magnetic resonance obtained pre-ablation, within 3 h and 3 months after procedures, in 41 patients with paroxismal AF undergoing PVI using PFA (18 patients) or thermal ablation (16 radiofrequency ablations and 7 cryoablations). In the acute stage, late gadolinium enhancement volume was 60% larger and oedema was 20% smaller after PFA compared to RF. Tissue modifications were more homogeneous after PFA than RF, without signs of microvascular damages or intramural haemorrhages. After 3 months, all the acute lesions disappeared after PFA and more lesions persisted after RF ablation. These data suggested a reparative process with a low grade of fibrosis in patients ablated using PFA and, moreover, this process might contribute to a preserved tissue compliance and left atrial function. 

Finally, although all safety data were reported, Gunawardene MA et al. [44] described the occurrence of coronary spasm during PFA of the mitral isthmus line. This kind of complication must be considered during mitral isthmus ablation.

## 4. Discussion

Pulse field ablation destabilizes cell membranes by forming irreversible pores, causing subsequent cell death. The electric field is most commonly produced by a high-voltage direct current delivered between two or more electrodes. Its nonthermal mechanism of ablation allows PFA to ablate atrial myocardium because it has a lower threshold for injury compared to the phrenic nerve and esophagus that are close to the heart. PFA also spares the extracellular matrix, preventing the disruption of tissue planes which characterizes RF damage (e.g., atrioesophageal fistula). However, this sparing of collateral organs is restricted to the clinical PFA doses studied, and these structures may be damaged if PFA is delivered at higher doses. 

Most AF ablation procedures are actually performed using RF, but this energy has many crucial factors to evaluate to create optimal transmural lesions, and over the years, new technologies have been developed to improve the quality of these lesions [8,9,10]. However, there are many limitations of current AF ablation procedures, and in particular, previous data reported a PV reconnection rate of 20% after PVI [12]. Regarding PFA settings, typical parameters include 10–90 pulses, with a pulse length of microseconds or nanoseconds (usually 100 ms), at a frequency of 1–10 Hz, and with an electric field between 500 and 3000 V/cm. In general, the greater the number of pulses, the higher the voltage, and the longer the pulse length, the more tissue is injured. However, there are other parameters to take into consideration for PFA. First, electroporation is dependent on cellular geometry and electrical field orientation, because the local current density creates an electrical field and the voltage across each tissue cell is proportional to its diameter measured in the axial direction of the electrical current [14,15,16,17,18,19,20,21,22,23,24,25,26,27,28,29,30,31,32,33,34,35]. For this reason, the electrode design is crucial to guarantee an optimal orientation of the electric field vector. Secondly, electroporation is susceptible to tissue properties because each cellular tissue may require different electrical field strengths to reach the eletroporation threshold. However, despite these factors needed to be taken into account to achieve a good lesion, all available data suggest that PFA might be more effective and safe than other technologies. 

All the animal studies reported data regarding the safety and efficacy of this new technology. In particular, the studies showed that PFA is effective in carrying out high-quality ablative lesions without injuring all the organs close to the heart. In particular, PFA has been performed on the PVs (both ostially and inside the vein), epicardium, endocardium, and also on the coronary vessels and their surrounding structures such as the phrenic nerve and esophagus [14,15,16,17,18,19,20,21,22,23,24,25,26,27,28,29,30,31,32,33,34,35]. However, the studies reported different types of electrode configurations and for this reason it will be important in the future to understand the best PFA energy to be effective and safe. 

The PFA advantages shown by animal studies have not only been demonstrated in preclinical evaluations, but also in clinical trial treating patients with paroxysmal and persistent AF. At the moment, most of the available clinical data regards Farapulse and Affera catheters [37,38], but other catheters are undergoing clinical evaluation for PFA. 

In patients with paroxysmal AF, PVI with PFA was safe, efficient, and durable with a low rate of recurrent atrial arrhythmias [36,37,38,39,40]. In particular, the studies reporting data using Farapulse [38] or Affera [39] catheters reported high efficacy in performing PVI with a low primary safety end point rate. Future results of the INSPIRE and PULSED AF trials [40,41] will give us other important information on this technology in the clinical practice. However, all the preliminary data of these two trials seem to confirm the efficacy and safety of this technology. 

In contrast to paroxysmal AF patients, in whom one-year success rates are reported as high as 90% [1,3], outcomes are significantly more modest in patients with persistent AF and patients who often require multiple procedures to maintain sinus rhythm. In patients with persistent AF, progressive structural and electrical remodeling creates a complex substrate for the initiation and maintenance of the arrhythmia and more extensive ablation strategies have been used, such as ablation of complex fractionated electrograms and rotor or voltage-based ablation [3]. During persistent AF ablation, the risk of procedural complications increases due to the higher number of lesions and, moreover, it is important to create durable lesions. Pulsed field ablation was also used to treat patients with persistent AF. A recent study [45] reported data regarding safety and lesion durability of PFA for both PVI and left atrial posterior wall (LAPW) ablation in persistent atrial fibrillation. In particular, acute PVI and LAPW ablation were successfully obtained in all patients; post-procedure esophagogastroduodenoscopy and repeat cardiac computed tomography revealed no mucosal lesions or PV narrowing, and invasive remapping demonstrated durable isolation (96% of PVI and 100% of LAPW isolation). 

The tissue-selective functionality of PFA is particularly relevant for atrial fibrillation ablation where complications such as phrenic palsy and esophageal injury are inexorably linked to strategies that aim to improve ablation efficacy. In particular, the LAPW has many anatomic and electrophysiologic characteristics that are important for the initiation and maintenance of persistent AF [46]. The LAPW wall is a potential target for ablation in patients with persistent AF because in this population, PVI alone has resulted in unsatisfactory recurrence rates [45]. Due to the anatomical proximity between the LAPW and the esophagus, the risk of esophageal damage is higher during persisent AF ablation [46,47]. The lack of any esophageal injury on endoscopic evaluation and the absence of phrenic nerve damage after PFA is important when considering this method as a valid strategy for performing persistent AF ablation. Moreover, recent studies using cardiac magnetic resonance have still confirmed the safety of PFA [41,42].

There are some issues to consider using PFA. The first is the necessity of general anesthesia and intubation before therapy delivery. In addition to the pain that the patient may feel, the main concern is skeletal muscle contraction that can create issues with catheter stability. A PFA delivery protocol named high-frequency irreversible electroporation (HFIRE), which uses multiple very brief pulses (down to 1 ms) at a high frequency (up to 1 MHz) in bipolar fashion, has been proposed to prevent muscle contractions [48,49]. Optimal sedation protocols are needed to perform PFA in order to reduce pain felt by patients, reduce muscle contractions, and, moreover, to avoid patient intubation. 

Finally, although PFA in animal studies was safe and effective, the optimal protocols and device delivery system are still unknown. In particular, some variables need to be considered, such as the amount of energy, number of pulses, pulse duration, and frequency. Moreover, devices and catheters used for PFA have some variables to consider such as surface area, amount of contact, or number of electrodes, and in the future it will be important to understand the most effective option to perform an effective and safe ablation.

## 5. Conclusions

Pulse field ablation is a new technology that seems to be effective and safe in treating patients affected by AF. All studies suggest that PFA is a promising tool for electrical PVI and myocardial ablation, resulting in excellent lesion durability and safety. Numerous experimental cardiac and non-cardiac studies demonstrate that most side effects and complications of thermal catheter ablation are not evident with electroporation ablation. Blood vessels, the esophagus, and nerves are spared in the midst of large electroporation lesions, and ablations deep inside the PV did not cause PV stenosis. 

## 6. Future Directions

At the moment, no studies have compared all the technologies to perform AF ablation, and even if PFA results show an excellent efficacy and safety, it is not possible to demonstrate a superiority of PFA compared to the other technologies. Further prospective studies will be needed to directly compare PFA to other technologies.

Moreover, electroporation is dependent on cellular geometry and electrical field orientation, and for this reason further studies are needed to demonstrate the optimal shape and electrode configuration to obtain a transmural and durable myocardial lesion using PFA. 

Furthermore, clinical data reporting a long-term follow up are needed to confirm the efficacy and safety of this new technology. 

Finally, due to PFA efficacy and safety, it will be interesting to evaluate clinical data regarding PFA for ventricular arrhythmias.

## Figures and Tables

**Table 1:** Most relevant preclinical irreversible electroporation (IRE) studies on cardiac tissue.

Study	Vitro/Vivo	Subject	Energy	Monopolar/Bipolar Electrode Configuration	Outcome and Side Effects
**Jian et al.** [22]	Vitro	HL-1 cell line	200 V; 1000 V/cm	Not specified	Effective lesions created
**Krassowska et al.** [23]	Vitro	Cardiac strand-2D model	0.4–0.5 V; 25 V/cm	Not specified	Pores in the first layer of cells
**Hirano et al.** [24]	Vivo	Porcine	Not specified	Bipolar	Healing process with preserved myocardial blood flow and little disruption of endocardium
**Lavee et al.** [25]	Vivo	Porcine	1500–2000 V	Not specified	Complete transmural destruction of atrial tissue and no local temperature change
**Du Pre et al.** [26]	Vivo	Porcine	Not specified	Not specified	One third of lesions was transmural, not damages to coronary arteries
**Zager et al.** [27]	Vivo	Rat	50, 250, 500 V	Not specified	Tissue damage related to pulse voltage
**Semenov et al.** [28]	Vivo	Rat	20 kV; 36 kV/cm	Not specified	Smaller pore size
**Sugrue et al.** [29]	Vivo	Canine	750 V	Bipolar	Minimal collateral damage to myocardium
**Al-Khadra et al.** [30]	Vivo	Rabbit	50–500 V	Bipolar	IRE might transiently reduce myocardial vulnerability to arrhythmias
**Stewart et al.** [31]	Vivo	Porcine	500 V	Bipolar	Lesions comparable to radiofrequency lesions and had no collateral damage
**Neven et al.** [32]	Vivo	Porcine	Not specified	Not specified	Thirty-one percent of lesions were transmural. No long-term luminal narrowing was seen.
**Hong et al.** [33]	Vivo	Ovine	Not specified	Bipolar	Well-demarcated lesions
**Padmanabhan et al.** [34]	Vivo	Canine	1000 V	Bipolar	Preservation of atrial myocardial architecture and absence of inflammatory reaction and fibrosis.
**Wittkampf et al.** [35]	Vivo	Porcine	950–2150 V	Monopolar	Lesion would be sufficient for inducing pulmonary vein isolation.

## Data Availability

Not applicable.

## References

[1] Hindricks G., Potpara T., Dagres N., Arbelo E., Bax J.J., Blomström-Lundqvist C., Boriani G., Castella M., Dan G.A., ESC Scientific Document Group (2021). 2020 ESC Guidelines for the diagnosis and management of atrial fibrillation developed in collaboration with the European Association for Cardio-Thoracic Surgery (EACTS): The Task Force for the diagnosis and management of atrial fibrillation of the European Society of Cardiology (ESC) Developed with the special contribution of the European Heart Rhythm Association (EHRA) of the ESC. Eur. Heart J..

[2] Wang T.J., Larson M.G., Levy D., Vasan R.S., Leip E.P., Wolf P.A., D’Agostino R.B., Murabito J.M., Kannel W.B., Benjamin E.J. (2003). Temporal relations of atrial fibrillation and congestive heart failure and their joint influence on mortality: The Framingham Heart Study. Circulation.

[3] Calkins H., Hindricks G., Cappato R., Kim Y.H., Saad E.B., Aguinaga L., Akar J.G., Badhwar V., Brugada J., Camm J. (2018). 2017 HRS/EHRA/EACS/APHRS/SOLAECE expert consensus statement on catheter and surgical ablation of atrial fibrillation. Europace.

[4] Schmidt B., Brugada J., Arbelo E., Laroche C., Bayramova S., Bertini M., Letsas K.P., Pison L., Romanov A., AFA LT Investigators Group (2020). Ablation strategies for different types of atrial fibrillation in Europe: Results of the ESC-EORP EHRA Atrial Fibrillation Ablation Long-Term registry. Europace.

[5] Pelargonio G., Di Monaco A., Guida P., Pellegrino P.L., Vergara P., Grimaldi M., Narducci M.L., Tritto M. (2022). AIAC Task Force AF Ablation. Atrial fibrillation ablation: Is common practice far from guidelines’ world? The Italian experience from a national survey. J. Interv. Card. Electrophysiol..

[6] Mont L., Bisbal F., Hernández-Madrid A., Pérez-Castellano N., Viñolas X., Arenal A., Arribas F., Fernández-Lozano I., Bodegas A., Cobos A. (2014). SARA investigators. Catheter ablation vs. antiarrhythmic drug treatment of persistent atrial fibrillation: A multicentre, randomized, controlled trial (SARA study). Eur. Heart J..

[7] Arbelo E., Brugada J., Hindricks G., Maggioni A.P., Tavazzi L., Vardas P., Laroche C., Anselme F., Inama G., Jais P. (2014). Atrial Fibrillation Ablation Pilot Study Investigators. The atrial fibrillation ablation pilot study: A European Survey on Methodology and results of catheter ablation for atrial fibrillation conducted by the European Heart Rhythm Association. Eur. Heart J..

[8] Kurose J., Kiuchi K., Fukuzawa K., Takami M., Mori S., Suehiro H., Nagamatsu Y.I., Akita T., Takemoto M., Yatomi A. (2020). Lesion characteristics between cryoballoon ablation and radiofrequency ablation with a contact force-sensing catheter: Late-gadolinium enhancement magnetic resonance imaging assessment. J. Cardiovasc. Electrophysiol..

[9] Stabile G., Di Donna P., Schillaci V., Di Monaco A., Iuliano A., Caponi D., Urraro F., Solimene F., Grimaldi M., Scaglione M. (2017). Safety and efficacy of pulmonary vein isolation using a surround flow catheter with contact force measurement capabilities: A multi center registry. J. Cardiovasc. Electrophysiol..

[10] Dello Russo A., Fassini G., Conti S., Casella M., Di Monaco A., Russo E., Riva S., Moltrasio M., Tundo F., De Martino G. (2016). Analysis of catheter contact force during atrial fibrillation ablation using the robotic navigation system: Results from a randomized study. J. Interv. Card. Electrophysiol..

[11] Andrade J.G., Champagne J., Dubuc M., Deyell M.W., Verma A., Macle L., Leong-Sit P., Novak P., Badra-Verdu M., Sapp J. (2019). CIRCA-DOSE Study Investigators. Cryoballoon or Radiofrequency Ablation for Atrial Fibrillation Assessed by Continuous Monitoring: A Randomized Clinical Trial. Circulation.

[12] Kuck K.H., Hoffmann B.A., Ernst S., Wegscheider K., Treszl A., Metzner A., Eckardt L., Lewalter T., Breithardt G., Willems S. (2016). Gap-AF–AFNET 1 Investigators*. Impact of complete versus incomplete circumferential lines around the pulmonary veins during catheter ablation of paroxysmal atrial fibrillation: Results from the Gap-Atrial Fibrillation-German Atrial Fibrillation Competence Network 1 Trial. Circ. Arrhythm. Electrophysiol..

[13] Yahsaly L., Siebermair J., Wakili R. (2022). Catheter ablation: Developments and technique selection. Herzschrittmacherther Elektrophysiol..

[14] Ye X., Liu S., Yin H., He Q., Xue Z., Lu C., Su S. (2021). Study on Optimal Parameter and Target for Pulsed-Field Ablation of Atrial Fibrillation. Front. Cardiovasc. Med..

[15] Avazzadeh S., O’Brien B., Coffey K., O’Halloran M., Keane D., Quinlan L.R. (2021). Establishing Irreversible Electroporation Electric Field Potential Threshold in A Suspension In Vitro Model for Cardiac and Neuronal Cells. J. Clin. Med..

[16] Song Y., Zheng J., Fan L. (2021). Nonthermal Irreversible Electroporation to the Esophagus: Evaluation of Acute and Long-Term Pathological Effects in a Rabbit Model. J. Am. Heart Assoc..

[17] Sugrue A., Vaidya V., Witt C., DeSimone C.V., Yasin O., Maor E., Killu A.M., Kapa S., McLeod C.J., Miklavčič D. (2019). Irreversible electroporation for catheter-based cardiac ablation: A systematic review of the preclinical experience. J. Interv. Card. Electrophysiol..

[18] Hunter D.W., Kostecki G., Fish J.M., Jensen J.A., Tandri H. (2021). In Vitro Cell Selectivity of Reversible and Irreversible: Electroporation in Cardiac Tissue. Circ. Arrhythm. Electrophysiol..

[19] Yavin H., Shapira-Daniels A., Barkagan M., Sroubek J., Shim D., Melidone R., Anter E. (2020). Pulsed Field Ablation Using a Lattice Electrode for Focal Energy Delivery: Biophysical Characterization, Lesion Durability, and Safety Evaluation. Circ. Arrhythm. Electrophysiol..

[20] Koruth J., Kuroki K., Iwasawa J., Enomoto Y., Viswanathan R., Brose R., Buck E.D., Speltz M., Dukkipati S.R., Reddy V.Y. (2019). Preclinical Evaluation of Pulsed Field Ablation: Electrophysiological and Histological Assessment of Thoracic Vein Isolation. Circ. Arrhythm. Electrophysiol..

[21] Yavin H., Brem E., Zilberman I., Shapira-Daniels A., Datta K., Govari A., Altmann A., Anic A., Wazni O., Anter E. (2021). Circular Multielectrode Pulsed Field Ablation Catheter Lasso Pulsed Field Ablation: Lesion Characteristics, Durability, and Effect on Neighboring Structures. Circ. Arrhythm. Electrophysiol..

[22] Jiang C., Goff R., Patana-anake P., Iaizzo P.A., Bischof J. (2013). Irreversible electroporation of cardiovascular cells and tissues. J. Med. Devices.

[23] Krassowska W. (1995). Effects of Electroporation on Transmembrane Potential Induced by Defibrillation Shocks. Pacing Clin. Electrophysiol..

[24] Hirano M., Yamamoto H., Hasebe Y., Fukuda K., Morosawa S., Amamizu H., Ohyama K., Uzuka H., Takayama K., Shimokawa H. (2018). Development of a Novel Shock Wave Catheter Ablation system—A Validation Study in Pigs in Vivo. Europace.

[25] Lavee J., Onik G., Rubinsky B. (2007). A novel nonthermal energy source for surgical epicardial atrial ablation: Irreversible electroporation. Heart Surg. Forum..

[26] Du Pre B.C., van Driel V.J., van Wessel H., Loh P., Doevendans P.A., Goldschmeding R., Wittkampf F.H., Vink A. (2013). Minimal Coronary Artery Damage by Myocardial Electroporation Ablation. Europace.

[27] Zager Y., Kain D., Landa N., Leor J., Maor E. (2016). Optimization of irreversible electroporation protocols for in-vivo myocardial decellularization. PLoS ONE.

[28] Semenov I., Zemlin C., Pakhomova O.N., Xiao S., Pakhomov A.G. (2015). Diffuse, non-polar electro permeabilization and reduced propidium uptake distinguish the effect of nanosecond electric pulses. Biochim. Biophys. Acta.

[29] Sugrue A., Vaidya V.R., Livia C., Padmanabhan D., Abudan A., Isath A., Witt T., DeSimone C.V., Stalboerger P., Kapa S. (2020). Feasibility of selective cardiac ventricular electroporation. PLoS ONE.

[30] Al-Khadra A., Nikolski V., Efimov I.R. (2000). The role of electroporation in defibrillation. Circ. Res..

[31] Stewart M.T., Haines D.E., Verma A., Kirchhof N., Barka N., Grassl E., Howard B. (2019). Intracardiac pulsed field ablation: Proof of feasibility in a chronic porcine model. Heart Rhythm.

[32] Neven K., Van Driel V., Van Wessel H., Van Es R., Doevendans P.A., Wittkampf F. (2014). Epicardial linear electroporation ablation and lesion size. Heart Rhythm.

[33] Hong J., Stewart M.T., Cheek D.S., Francischelli D.E., Kirchhof N. Cardiac ablation via electroporation. Proceedings of the 31st Annual International Conference of the IEEE Engineering in Medicine and Biology Society: Engineering the Future of Biomedicine, EMBC 2009.

[34] Padmanabhan D., Naksuk N., Killu A.K., Kapa S., Witt C., Sugrue A., DeSimone C.V., Madhavan M., de Groot J.R., O’Brien B. (2019). Electroporation of epicardial autonomic ganglia: Safety and efficacy in medium-term canine models. J. Cardiovasc. Electrophysiol..

[35] Wittkampf F.H.M., Van Driel V.J., Van Wessel H., Neven K.G.E.J., Gründeman P.F., Vink A., Loh P., Doevendans P.A. (2012). Myocardial lesion depth with circular electroporation ablation. Circ. Arrhythm. Electrophysiol..

[36] Reddy V.Y., Koruth J., Jais P., Petru J., Timko F., Skalsky I., Hebeler R., Labrousse L., Barandon L., Kralovec S. (2018). Ablation of atrial fibrillation with pulsed electric fields: An ultra-rapid, tissue-selective modality for cardiac ablation. JACC Clin. Electrophysiol..

[37] Reddy V.Y., Neuzil P., Koruth J.S., Petru J., Funosako M., Cochet H., Sediva L., Chovanec M., Dukkipati S.R., Jais P. (2019). Pulsed Filed Ablation for Pulmonary Vien Isolation in Atrial Fibrillation. J. Am. Coll. Cardiol..

[38] Reddy V.Y., Dukkipati S.R., Neuzil P., Anic A., Petru J., Funasako M., Cochet H., Minami K., Breskovic T., Sikiric I. (2021). Pulsed Field Ablation of Paroxysmal Atrial Fibrillation: 1-Year Outcomes of IMPULSE, PEFCAT, and PEFCAT II. JACC Clin. Electrophysiol..

[39] Reddy V.Y., Anter E., Rackauskas G., Peichl P., Koruth J.S., Petru J., Funasako M., Minami K., Natale A., Jais P. (2020). Lattice-Tip Focal Ablation Catheter That Toggles Between Radiofrequency and Pulsed Field Energy to Treat Atrial Fibrillation: A First-in-Human Trial. Circ. Arrhythm. Electrophysiol..

[40] De Potter T., Reddy V., Neuzil P., Rackauskas G., Anic A., Grimaldi M., Di Biase L., Natale A. (2021). Acute safety and performance outcomes from the inspIRE trial using a novel pulsed field ablation system for the treatment of paroxysmal atrial fibrillation. Eur. Heart J..

[41] Verma A., Boersma L., Haines D.E., Natale A., Marchlinski F.E., Sanders P., Calkins H., Packer D.L., Hummel J., Onal B. (2022). First-in-Human Experience and Acute Procedural Outcomes Using a Novel Pulsed Field Ablation System: The PULSED AF Pilot Trial. Circ. Arrhythm. Electrophysiol..

[42] Cochet H., Nakatani Y., Sridi-Cheniti S., Cheniti G., Ramirez F.D., Nakashima T., Eggert C., Schneider C., Viswanathan R., Derval N. (2021). Pulsed field ablation selectively spares the oesophagus during pulmonary vein isolation for atrial fibrillation. Europace.

[43] Nakatani Y., Sridi-Cheniti S., Cheniti G., Ramirez F.D., Goujeau C., André C., Nakashima T., Eggert C., Schneider C., Viswanathan R. (2021). Pulsed field ablation prevents chronic atrial fibrotic changes and restrictive mechanics after catheter ablation for atrial fibrillation. Europace.

[44] Gunawardene M.A., Schaeffer B.N., Jularic M., Eickholt C., Maurer T., Akbulak R.Ö., Flindt M., Anwar O., Hartmann J., Willems S. (2021). Coronary Spasm During Pulsed Field Ablation of the Mitral Isthmus Line. JACC Clin. Electrophysiol..

[45] Reddy V.Y., Anic A., Koruth J., Petru J., Funasako M., Minami K., Breskovic T., Sikiric I., Dukkipati S.R., Kawamura I. (2020). Pulsed Field Ablation in Patients with Persistent Atrial Fibrillation. J. Am. Coll. Cardiol..

[46] Terricabras M., Piccini J.P., Verma A. (2020). Ablation of persistent atrial fibrillation: Challenges and solutions. J. Cardiovasc. Electrophysiol..

[47] Di Monaco A., Quadrini F., Katsouras G., Caccavo V., Troisi F., Quatraro F., Cecere G., Langialonga T., Grimaldi M. (2015). Ablation of atrial fibrillation and esophageal injury: Role of bipolar and unipolar energy using a novel multipolar irrigated ablation catheter. Heart Rhythm.

[48] Arena C.B., Sano M.B., Rossmeisl J.H., Caldwell J.L., Garcia P.A., Rylander M.N., Davalos R.V. (2011). High-frequency irreversible electroporation (H-FIRE) for non-thermal ablation without muscle contraction. Biomed. Eng. Online.

[49] Sano M.B., Fan R.E., Xing L. (2017). Asymmetric Waveforms Decrease Lethal Thresholds in High Frequency Irreversible Electroporation Therapies. Sci. Rep..

